# Mixed functional tumor of the left adrenal as a cause of palpitations, surgical management. Case report

**DOI:** 10.1093/jscr/rjae731

**Published:** 2024-11-26

**Authors:** Santiago Muñoz-Palomeque, William Aguayo-Vistin, Gabriel A Molina, Zanny Bastidas-Arévalo, Jaime Paul Herrera Gonzalez, Christian I Gordon

**Affiliations:** PGY3, General and Laparoscopic Surgery, Universidad Internacional del Ecuador, Quito 170411, Ecuador; General Surgery Department, Hospital Metropolitano, Quito 170508, Ecuador; Department of Surgery, Division of General Surgery, Hospital General San Francisco, Quito 170120, Ecuador; Universidad San Francisco de Quito, Department of General Surgery, Hospital Metropolitano, Quito 170508, Ecuador; PGY1, Internal Medicine, Universidad Internacional del Ecuador, Quito 170411, Ecuador; Department of Internal Medicine, Hospital Metropolitano, Quito 170508, Ecuador; Department of Internal Medicine, Hospital Metropolitano, Quito 170508, Ecuador; PGY3, General and Laparoscopic Surgery, Universidad Internacional del Ecuador, Quito 170411, Ecuador; General Surgery Department, Hospital Metropolitano, Quito 170508, Ecuador

**Keywords:** adrenal gland neoplasms, neuroblastoma, neoplasms, complex and mixed paraganglioma, pheochromocytoma

## Abstract

Compound pheochromocytoma refers to a rare adrenal tumor that includes neuroblastic components and is a rare catecholamine-producing tumor from chromaffin cells, typically found in the adrenal medulla. It usually presents with symptoms like tachycardia, headache, and intermittent diaphoresis, although its clinical manifestations can vary. Diagnosis involves biochemical studies and imaging such as catecholamines, metanephrines, CT scans, and positron emission tomography (PET). The surgical management is the definitive, being the laparoscopic approach of choice in most cases. This case report discusses a 45-year-old male who presented with tachycardia and palpitations, diagnosed with left pheochromocytoma exhibiting neuroblastoma differentiation, surgically treated through anterior laparoscopy without any trans-surgical complications and with low bleeding. Postoperative recovery was uneventful, and pathology confirmed the diagnosis. Timely diagnosis and surgical removal are crucial, with laparoscopy being the preferred approach for tumor resection.

## Introduction

Pheochromocytoma is a rare catecholamine-producing tumor originating from chromaffin cells of the sympathetic nervous system, occurring at an incidence of 0.8 cases per 100 000 annually [1]. Typically developing between the fourth and fifth decades of life [2], over 80% of these tumors are found in the adrenal medulla, with the rest arising in extra-adrenal chromaffin tissues, such as the para-aortic sympathetic ganglion chain or organs like Zuckerkandl's. When these tumors develop outside the adrenal glands, they are called paragangliomas [1].

Pheochromocytomas can be sporadic or linked to genetic syndromes such as multiple endocrine neoplasia type 2 (MEN2A and 2B), Von Hippel–Lindau disease, neurofibromatosis type 1, or familial paraganglioma [3].

## Case report

A 45-year-old male patient with no significant medical history who presented with a history of sinus tachycardia of 110 beats per minute, without additional symptoms, 2 months prior to admission for a medical check-up, complemented by 24-hour Holter studies, complete blood count, thyroid function and lipid profile within normal parameters. Then, he began experiencing frequent episodes of tachycardia and palpitations without hourly predominance exacerbated with physical activity. These were associated with cervical pain, occipital headache, hyporexia, and a 5-kilogram weight loss over 2 months.

**Figure 1:**
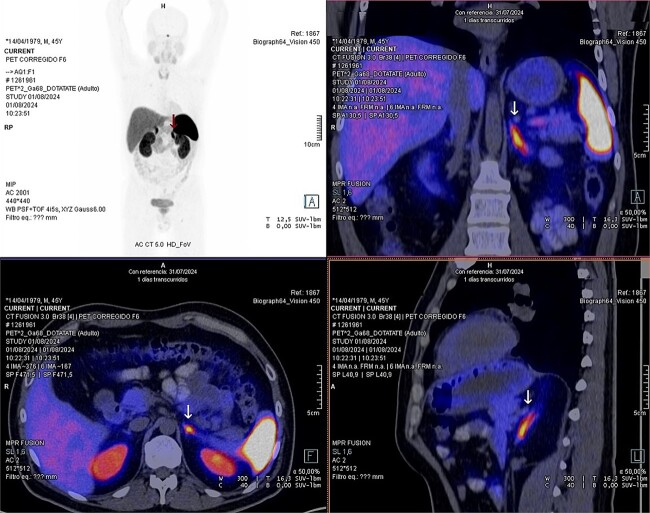
PET-SCAN with gadotatate study with the tumor capitation evidence (arrows).

Laboratory studies were complemented with elevated metanephrines in urine three times the normal value (3.19 mg/24 hours), and in a gadotatate positron emission tomography (PET-SCAN) study (Fig. 1), showed a slightly enlarged left adrenal gland with increased metabolic activity, leading to the diagnosis of left pheochromocytoma.

**Figure 2:**
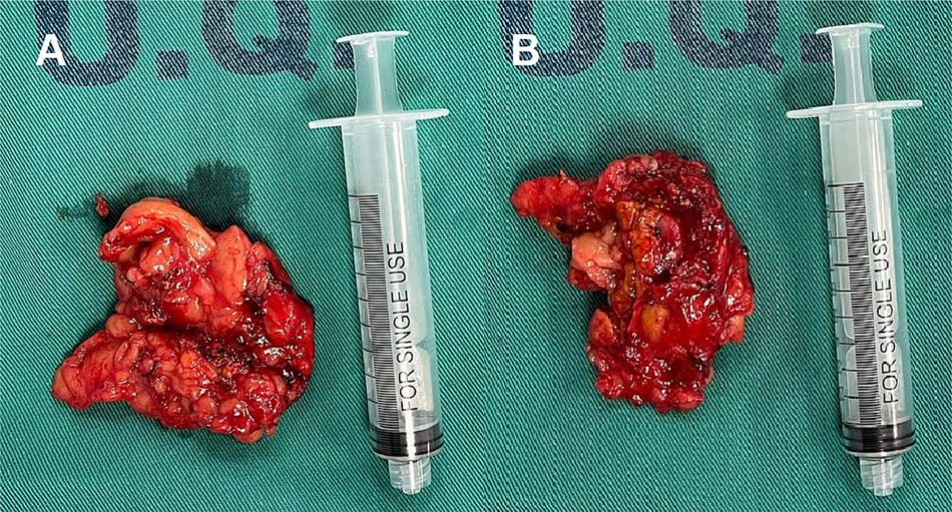
Left compound pheochromocytoma with neuroblastoma in differentiation.

Laparoscopic exploration revealed a left adrenal gland measuring 6x4x3 cm, adherent to retroperitoneum, spleen and ipsilateral kidney, with extension of the lower lobe of the same up to the height of the hilum and renal artery, with an inflammatory appearance and indurated consistency (Fig. 2), and a lymph node of 1 × 2 cm adhered to the renal hilum. A laparoscopic left adrenalectomy and regional lymphadenectomy were performed without complications. Postoperatively, the patient remained hemodynamically stable with no evidence of catecholamine crisis, and was discharged home at the second day. No further episodes of tachycardia or palpitations were reported during recovery.

**Figure 3:**
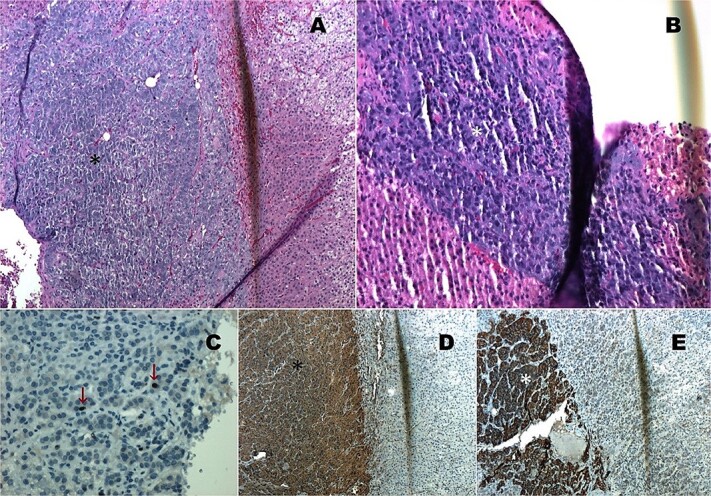
Histopathology images. (A) HE 4×: Compound pheochromocytoma (*). (B) Neuroblastoma in differentiation (*). (C) Ki67 proliferation index of 1% in the adrenal medullary area (*). (D) Immunohistochemistry with cytoplasmic chromogranin positivity (*). (E) Immunohistochemistry with cytoplasmic synaptophysin positivity (*).

Histopathological analysis confirmed a diagnosis of compound pheochromocytoma with neuroblastoma differentiation. The tumor showed no necrosis or vascular invasion, and the proliferation index (Ki67) was 1%. Immunohistochemical markers such as cytoplasmic chromogranin, synaptophysin, and PGP 9.5 were strongly positive in the adrenal medullary cells (Fig. 3).

## Discussion

Pheochromocytomas derive their name from the brown color of their granules, which stain with chromic acid due to the oxidation of catecholamines [2]. Compound pheochromocytomas, which include neuroblastic components, are rare and have been linked to shared genetic mutations, such as alterations in the FGFR1 gene [4–7]. These tumors carry a high risk of mortality if left untreated, with reported rates between 30% and 45%. However, the 5-year event-free survival after surgical treatment is 40%, and overall survival is about 63% [8, 9].

Clinically, pheochromocytomas are often recognized by the classic triad of symptoms: tachycardia, headache, and intermittent diaphoresis. These symptoms are present in over 90% of cases. About half of the patients present with arterial hypertension, while 10%–15% are normotensive [1]. However, pheochromocytomas can also mimic other conditions, such as endocrine, metabolic, or neurological disorders [10], as seen in this case where the patient initially presented with tachycardia and palpitations.

The diagnosis is confirmed by biochemical testing, including urine and plasma catecholamines, metanephrines, and vanillylmandelic acid. Elevated total metanephrines above 400 micrograms in 24 hours is highly suggestive of pheochromocytoma [3, 11, 12]. Imaging techniques, such as ultrasound, CT scans, MRI, or PET, are crucial for determining tumor location. In recent years, a combination of both has also been used, such as single photon emission computed tomography (SPECT/CT) or PET/CT [13]. CT is often the preferred imaging modality [11], with a sensitivity of over 95% for detecting adrenal tumors larger than 5–10 mm [13]. The features of pheochromocytoma on CT include a well-circumscribed adrenal nodular lesion that presents a high signal on T2, rapid and intense enhancement, and frequent cystic and hemorrhagic phenomena [14].

Advanced molecular imaging, such as 68Ga-DOTATATE PET scans, is particularly useful in identifying metastatic pheochromocytoma and paraganglioma. These tumors express somatostatin receptors SSTR2 and SSTR3, which 68Ga-DOTATATE binds to, allowing for precise imaging and evaluation of genetic mutations like SDHx [15].

Once diagnosed, surgery is the primary treatment. Laparoscopy is considered a safe and effective approach for adrenalectomy, with options including anterior, thoracoabdominal, or retroperitoneal routes depending on the tumor's location and size [16, 17]. Open surgery is typically reserved for tumors larger than 7–8 cm [18]. These surgeries carry significant risk due to the potential for hemodynamic instability, making a multidisciplinary team essential [16].

Intraoperatively, careful tumor manipulation is critical, as it can trigger the release of catecholamines, leading to dangerous spikes in blood pressure and increased bleeding. Controlling the operative field and ensuring complete resection without capsular rupture are important steps for a safe adrenalectomy [18].

In the case presented, a laparoscopic approach was successfully used, similar to previous studies where this method led to shorter recovery times and reduced postoperative pain. For instance, one study of a series of five successful laparoscopic adrenalectomies for various etiologies, involving a 33-year-old woman with bilateral pheochromocytomas showed no need for transfusions, with an average hospital stay of 3–4 days, concluding that this technique adequately removes adrenal tumors surgically and produces less postoperative pain with a faster recovery [17].

## Conclusions

Compound pheochromocytoma is a rare adrenal tumor with neuroblastic components that can cause excessive catecholamine production, leading to severe cardiovascular complications, such as arrhythmias, cardiac hypertrophy, cardiomyopathy, and even develop into heart failure. It also has an insidious presentation that can lead to diagnostic errors. Due to its varied presentation, timely diagnosis is crucial to prevent misdiagnosis and ensure prompt treatment.

Laparoscopic adrenalectomy remains the preferred treatment approach for such tumors, offering a minimally invasive method for complete tumor removal with fewer postoperative complications and quicker recovery times. The surgical approach should be tailored to each patient, considering tumor size, location, and malignancy risk.
